# High-BMI-related low back pain in China: a GBD-based observational study on sex–age trends and projections (1990–2021)

**DOI:** 10.1186/s40001-025-02765-3

**Published:** 2025-06-19

**Authors:** Juxiang Xu, Jianhua Li, Heqing Huang, Tiangao Lin, Zhiping Liao, Weibo Zhang, Jianing Wu, Fangchao Wu

**Affiliations:** 1 Nursing Department, Sir Run Run Shaw Hospital, Zhejiang University School of Medicine, Hangzhou, Zhejiang China; 2 Department of Physical Medicine and Rehabilitation, Sir Run Run Shaw Hospital, Zhejiang University School of Medicine, Hangzhou, China; 3 Center for Diagnosis, Treatment and Rehabilitation of Pelvic Floor Dysfunction, Sir Run Run Shaw Hospital, Zhejiang University School of Medicine, Hangzhou, China; 4 Department of Pain Medicine, Sir Run Run Shaw Hospital, Zhejiang University School of Medicine, Hangzhou, China

**Keywords:** Low back pain, High-BMI, Disease burden, Prediction, China, GBD

## Abstract

**Background:**

Low back pain (LBP), the leading global cause of disability, imposes severe burdens in China, where obesity (BMI ≥ 28 kg/m^2^) quadruples LBP risk. Despite concurrent obesity and LBP epidemics, secular trends of BMI-related disability remain unquantified.

**Objective:**

Using data from the Global Burden of Disease (GBD) 2021 study, we conducted an observational, cross-sectional analysis of three-decade trends (1990–2021) in disability due to high-BMI-related LBP across China, stratified by age and sex. By comparing national and global trajectories, we inform targeted strategies addressing this dual epidemic.

**Methods:**

GBD 2021 data informed age-standardized years lived with disability (YLD) analyses of BMI-linked LBP disability (1990–2021) in China. Joinpoint regression identified trend inflection points, with annual percentage change and average annual percentage change (APC/AAPC) calculated using R and Joinpoint software for age–sex stratified burden quantification. Bayesian age–period cohort (BAPC) model was used to estimate the trend of burden of low back pain attributable to high BMI from 2022 to 2036.

**Results:**

Between 1999 and 2021, China’s high-BMI-related LBP disability burden escalated disproportionately, with age-standardized YLD rates rising 116.5% versus 39.1% globally. Females exhibited persistently higher burdens (2021 age-standardized YLD rate (ASYR): 67.51 vs. male 37.45/100,000), while youth (< 30 years) saw accelerated male-dominant growth (20–24 years: 17.57% annual increase post 2015). Joinpoint analysis identified China’s unique triphasic acceleration (AAPC = 2.54%, *P* < 0.05), contrasting with linear global trends. Aging populations showed widening gender gaps (e.g., 60–64 years: female YLD rates 2.67-fold males), highlighting urgent need for sex- and age-tailored interventions.

**Conclusion:**

China's high-BMI-related LBP burden, though currently below global levels, is escalating disproportionately—accelerating in young males and persistently higher in females. Prioritizing sex- and age-specific interventions is critical to curb this dual metabolic–mechanical epidemic.

*Trial Registration*: This study was an observational study analysis based on the GBD database, and no clinical trial registration was performed.

**Supplementary Information:**

The online version contains supplementary material available at 10.1186/s40001-025-02765-3.

## Introduction

Low back pain (LBP), though nonfatal, has emerged as a defining global health crisis of the twenty-first century, reigning as the foremost cause of disability worldwide and unleashing catastrophic socioeconomic repercussions [1]. The epidemic's trajectory is alarmingly exponential: in 2020, 619 million lives were shackled by LBP globally, a figure projected to skyrocket to 843 million by 2050—fueled by an aging tsunami and a pandemic of sedentary lifestyles [2]. China stands at the epicenter of this silent catastrophe, confronting unparalleled epidemiological severity. In 2019, the nation bore the heaviest global burden of LBP, with 91.3 million afflicted individuals and 8.6 million years of healthy YLDs [3]—a stark testament to LBP's unchallenged dominance as the archvillain of disability in the country.

Accumulating empirical evidence substantiates elevated body mass index (BMI) as a pivotal modifiable risk factor in the multifactorial pathogenesis of LBP [4, 5]. Biomechanically, excess adiposity imposes supraphysiological axial loading on spinal structures, directly amplifying intervertebral disc pressure and precipitating accelerated degenerative cascades [6]. Biologically, adipocytokine dysregulation—particularly leptin and adiponectin imbalance—induces profound systemic inflammation and metabolic derangements, creating a microenvironment conducive to chronic nociceptive signaling and tissue remodeling [7, 8]. Furthermore, insulin resistance and vascular endothelial dysfunction act synergistically to compromise nutrient diffusion to avascular intervertebral discs; thereby, exacerbating hypoxia-driven matrix degradation and cellular apoptosis [9]. Critically, emerging evidence highlights that spinal epidural lipomatosis (SEL) mediates the association between elevated BMI and low back pain in affected individuals. SEL manifests as pathological epidural fat accumulation within the spinal canal in high-BMI patients, compressing nerve roots or the dural sac, thereby inducing spinal stenosis and pain [10]. MRI-based studies report a 15–24% prevalence of SEL in obese cohorts, with epidural fat volume increasing by ~ 45 mm^3^ per 1-unit BMI increment [11]. Postbariatric surgery reductions in epidural fat volume (~ 40%) and concomitant significant pain relief conclusively confirm the causal role of SEL in BMI-related low back pain [12].

Globally, obesity (BMI ≥ 30 kg/m^2^) is a well-documented risk amplifier for LBP, with a meta-analysis of 33 studies reporting 1.53-fold higher incidence in obese populations as compared to normal-weight individuals [13]. Notably, this association escalates dramatically in China—a nation undergoing rapid nutritional transition. Li's meta-analysis revealed a 4.51-fold surge in LBP risk among Chinese individuals with BMI ≥ 28 kg/m^2^ [14], a threshold lower than the global obesity definition yet associated with disproportionately severe consequences. Alarmingly, China now shoulders a dual burden: it leads the world with 40 million obese adults as of 2021 [15], while simultaneously ranking first in LBP-related disability. Despite these converging crises, critical evidence gaps persist in quantifying the secular trends of BMI-attributable LBP disability burden, hindering targeted policy interventions.

China faces an accelerating dual epidemic of obesity and disabling LBP, yet critical gaps persist in understanding how gender, age, and temporal trends modulate the disability burden attributable to high BMI. To address this urgent public health challenge, our study leverages comprehensive data from the Global Burden of Disease Study 2021, systematically examining three-decade trajectories (1990–2021) of BMI-related LBP disability across China. Through gender- and age-stratified analyses—and by contrasting national patterns with global benchmarks—we unmask previously unrecognized epidemiological heterogeneities. BAPCmodel was used to estimate the trend of burden of low back pain attributable to high BMI from 2022 to 2036. These findings provide actionable evidence to prioritize high-risk subgroups and design precision interventions against this escalating syndemic.

## Materials and methods

### Data source

Epidemiological data were sourced from the Global Burden of Diseases, Injuries, and Risk Factors Study (GBD) 2021 database (accessed March 8, 2025, via http://ghdx.healthdata.org/gbd-results-tool). GBD data processing followed GBD standardized pipelines, including three-tier quality controls: (1) outlier detection using interquartile range filtering, (2) geospatial bias correction through Gaussian process regression, and (3) cross-walk standardization for heterogeneous diagnostic criteria across data sources. This study represents a secondary observational analysis of the Global Burden of Disease (GBD) 2021 dataset. We conducted a cross-sectional assessment of global disease burden patterns for the reference year 2021, combined with time–trend analyses (1990–2021) to evaluate longitudinal changes. Statistical modeling followed the standardized GBD framework, utilizing DisMod-MR for data synthesis and uncertainty estimation [16]. LBP was defined according to GBD criteria as pain localized to the posterior region extending from the inferior margin of the twelfth ribs to the lower gluteal folds, with or without radiation to the lower limbs, persisting for ≥ 1 day. Adults aged ≥ 20 years were classified as having elevated BMI using population-specific thresholds: > 23 kg/m^2^ for Asian populations and > 25 kg/m^2^ for non-Asian populations, as per GBD risk assessment protocols [16, 17].

YLD were utilized to quantify nonfatal disease burden, calculated via the formula:

YLD = Prevalence × Disability Weight (DW) × Mean Duration.

DW values, stratified by LBP severity levels according to GBD criteria [18], range from 0 (no health loss) to 1 (health loss equivalent to death). We analyzed global YLD estimates attributable to high-BMI-associated LBP from 1990 to 2021. Individuals aged < 20 years were excluded due to negligible YLD contributions from high-BMI-related LBP within this age group.

### Descriptive analysis

Temporal trends in age-standardized YLD rates were evaluated using three complementary analytical metrics:

#### Simple percentage change

The absolute percentage change in YLD rates between the initial (1990) and final (2021) years of the study period.

$${\text{SPC}} = \frac{{\left( {{\text{Final YLDs}} - {\text{Initial YLDs}}} \right)}}{{\text{Initial YLDs}}} \times 100{\text{\% }}$$

#### Annual percent change (APC) [19]

Derived from segmented log-linear regression models, the APC quantifies the yearly rate of change in YLD rates within discrete temporal intervals. The slope coefficient (β) of the regression equation represents the natural logarithm-transformed APC:

$${\text{APC}} = \left( {{\text{e}}^{{\upbeta }} - 1} \right) \times 100{\text{\% }}$$

#### Average annual percentage change (AAPC)

A weighted average of APC across all segments identified by Joinpoint regression, calculated as:

$${\text{AAPC}} = \left[ {\left( {\mathop \prod \limits_{i = 1}^{n} \left( {1 + \frac{{{\text{APC}}_{i} }}{100}} \right)^{{w_{i} }} } \right)^{\frac{1}{T}} - 1} \right] \times 100\%$$

where.

*n*: Total number of segments identified by the Joinpoint regression model;

*wi*: Duration (years) of the *i*th segment, serving as a weight to account for heterogeneous interval lengths;

*T:* Total study duration (32 years).

### Joinpoint regression analysis

Joinpoint regression analysis is a segmented linear regression methodology designed to detect inflection points (joinpoints) in temporal trends where significant shifts in trajectory occur. The model employs likelihood ratio tests to statistically evaluate the significance of slope parameters, expressed as APC. The AAPC is subsequently computed as a weighted geometric mean of APCs across identified segments, providing a consolidated measure of overall trend magnitude during the study period [19].

Analyses were performed using Joinpoint Regression Software 4.9.1.0 (National Cancer Institute, Rockville, MD, USA) to detect joinpoints in the temporal burden of LBP attributable to high BMI. APC and AAPC were computed to assess direction and magnitude of trends. A significant increasing trend was defined as APC > 0 with a 95% confidence interval (CI) excluding 0; conversely, APC < 0 with CI excluding 0 indicated a significant decreasing trend. Nonsignificant trends (*P* ≥ 0.05) were identified if CIs included 0. For multisegment trends, AAPC was derived by weighting segment-specific APCs by their duration, reflecting the compounded annualized trend.

### BAPC model

The BAPC model extends the traditional APC framework by incorporating stochastic smoothness priors and dynamic population structures. To study future trends in the burden of low back pain caused by high BMI, we projected changes in age-standardized YLDs from high-BMI-induced low back pain between 2022 and 2036, and performed subgroup analyses by sex. Baseline population data were obtained from the GBD study’s age-stratified population records spanning 1990 to 2021. Population projections for 2022–2036 were uniformly calibrated according to the GBD standard population structure. Analyses utilized the BAPC and integrated nested Laplace approximation (INLA) packages in R statistical software, with the efficient INLA algorithm replacing Markov chain Monte Carlo simulations (MCMC) [20].

### Statistical analysis

All statistical analyses were performed using R software (version 4.4.2) integrated with the GBD Results Tool Kit and Joinpoint Regression Software (version 4.9.1.0) to ensure methodological consistency in data standardization. Age-standardized rates were calculated through direct standardization against the GBD 2021 reference population structure. The 95% (Uncertainty Interval) UI are employed to represent YLDs and YLDs rate. The 95% UIs of the final burden estimates were calculated by producing 500–1000 draws of the posterior distribution at every modelling step, carrying out draw-level calculations for any subsequent scaling, and reporting the draws corresponding to the 2·5th percentile and 97·5th percentile of the distribution for each quantity reported [21]. The 95% Confidence Interval (CI) was calculated based on the standard error (SE) and the central limit theorem [22]. It is designed to describe the estimated range of the calculated APC, AAPC, and data in the BAPC model. Statistical significance was evaluated using two-tailed tests with a predefined alpha level of 0.05. The study employed de-identified, population-level data from the GBD repository, which adheres to Article 32 of the Helsinki Declaration governing the ethical use of secondary epidemiological data.

## Results

### Trend of disease burden of low back pain related to high BMI (1990–2021)

Table 1 presents a comprehensive comparison of DALYs and ASDR by GBD regions over this period, including AAPC values. Overall, both China and the global population exhibited increasing trends in both the absolute number and age-standardized rates of YLDs attributable to BMI-related LBP during this period, with China demonstrating a higher ASYR growth rate as compared to the global average.

**Table 1:** All-age cases and age-standardized YLDs for LBP attributable to BMI in global, super region and China: 1990 vs. 2021

Location	1990		2021		1990–2021
	All-ages YLDs cases	Age-standardized YLDs rate (per 100,000)	All-ages YLDs cases	Age-standardized YLDs rate (per 100,000)	AAPC of Age-standardized YLDs rate
	(95% UI)	/10^5^ (95% UI)	(95% UI)	/10^5^ (95% UI)	(95% CI)
Global	3086573 (312559, 6484427)	70.22 (7.14, 146.48)	8363759 (840307, 17424822)	97.66 (9.78, 204.00)	1.07 (1.06, 1.09)
Female	2,072,340 (207877, 4368723)	92.01 (9.25, 193.20)	5,541,374 (556566, 11636895)	126.29 (12.63, 266.01)	1.03 (1.01, 1.05)
Male	1,014,233 (104683, 2115704)	46.57 (4.84, 96.25)	2,822,385 (283741, 5786405)	67.56 (6.78, 138.87)	1.21 (1.19, 1.23)
China	245,216 (29997, 500110)	24.34 (2.99, 49.33)	1,062,018 (109971, 2225149)	52.70 (5.41, 110.10)	2.54 (2.46, 2.62)
Female	154,793 (18912, 315730)	31.60 (3.87, 64.09)	703,298 (73131, 1478285)	67.51 (6.96, 142.32)	2.51 (2.46, 2.55)
Male	90,423 (11086, 186919)	17.12 (2.12, 35.11)	358,719 (36840, 745322)	37.45 (3.81, 77.55)	2.55 (2.52, 2.59)
Super regions					
Central Europe, Eastern Europe, and Central Asia	737551 (71921,1547897)	157.47 (15.31,329.58)	1168137 (117798,2408121)	200.95 (19.94,415.25)	0.79 (0.78, 0.81)
High income	1272168 (124875,2651915)	118.15 (11.52,246.82)	2495269 (250649,5135704)	163.3 (16.11,336.14)	1.04 (1.02, 1.07)
Latin America and Caribbean	248302 (24319,524844)	87.9 (8.7,184.56)	905799 (88653,1876505)	141.1 (13.81,293.14)	1.54 (1.52, 1.55)
North Africa and Middle East	230949 (22552,487952)	104.86 (10.41,219.7)	1037525 (106623,2092696)	179.03 (18.31,358.27)	1.74 (1.73, 1.76)
South Asia	151159 (16626,302155)	19.73 (2.19,39.35)	796921 (76445,1635332)	46.11 (4.48,94.36)	2.79 (2.74, 2.84)
Southeast Asia, East Asia, and Oceania	328163 (39130,679716)	23.74 (2.85,48.89)	1460703 (149004,3067737)	51.75 (5.25,108.55)	2.55 (2.51, 2.59)
Sub-Saharan Africa	118282 (12538,240805)	43.46 (4.66,87.86)	499405 (49110,1041058)	74.89 (7.57,154.72)	1.77 (1.76,1.78)

*YLD* Years Lived with Disability, *LBP* Low Back Pain, *BMI* Body Mass Index, *AAPC* Average Annual Percent Change, *UI* Uncertainty Interval, *CI* Confidence Interval

In China, the age-standardized DALY rate increased by 116.49%, rising from 24.34 per 100,000 population (95% UI: 2.99–49.33) in 1990 to 52.70 per 100,000 (95% UI: 5.41–110.10) in 2021. Stratified by gender, females experienced an increase from 31.60 per 100,000 (95% UI: 3.87–64.09) to 67.51 per 100,000 (95% UI: 6.96–142.32; 113.64% increase), while males increased from 17.12 per 100,000 (95% UI: 2.12–35.11) to 37.45 per 100,000 (95% UI: 3.81–77.55; 118.68% increase). Concurrently, the absolute number of YLDs surged from 245,216 (95% UI: 29,997–500,110) in 1990 to 1,062,018 (95% UI: 109,971–2,225,149) in 2021, with increases observed across both genders.

Globally, the ASYR for BMI-related LBP increased by 39.07%, from 70.22 per 100,000 (95% UI: 7.14–146.48) in 1990 to 97.66 per 100,000 (95% UI: 9.78–204.00) in 2021. Among females, the rate rose from 92.01 per 100,000 (95% UI: 9.25–193.20) to 126.29 per 100,000 (95% UI: 12.63–266.01; 37.26% increase), while males showed an increase from 46.57 per 100,000 (95% UI: 4.84–96.25) to 67.56 per 100,000 (95% UI: 6.78–138.87; 45.08% increase). The global YLD count also escalated from 3,086,573 (95% UI: 312,559–6,484,427) in 1990 to 8,363,759 (95% UI: 840,307–17,424,822) in 2021.

During the period 1990–2021, Central/Eastern Europe and Central Asia exhibited the highest burden rate (age-standardized YLDs rate in 2021: 200.95 per 100,000 [95% UI: 19.94–415.25]), albeit with the modest growth (AAPC 0.79% [95% CI 0.78–0.81]). In contrast, South Asia (46.11 per 100,000 [4.48–94.36]) and Southeast Asia/East Asia/Oceania (51.75 per 100,000 [5.25–108.55]) demonstrated comparatively lower the current burdens but showed the most rapid global increases (AAPC 2.79% [2.74–2.84] and 2.55% [2.51–2.59], respectively). High-income regions recorded the highest absolute all-age YLDs count (2,495,269 cases [95% UI: 250,649–5,135,704]), while simultaneously maintaining the second-highest age-standardized rate (163.30 per 100,000 [16.11–336.14]).

### Differences in YLD trends between China and the world (1990–2021)

**Fig. 1:**
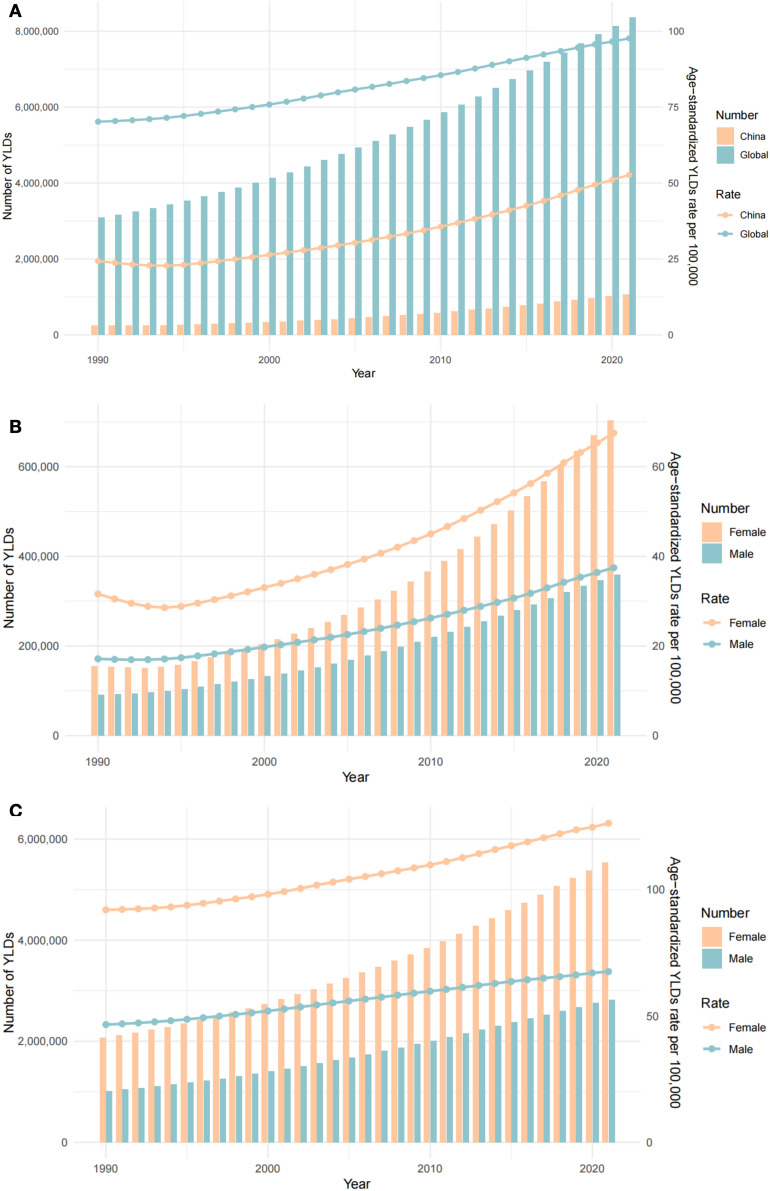
Differences in YLD trends between China and the world (1990–2021). **A** Comparative analysis of YLD and ASYR trends between China and the world. **B** Gender-stratified analyses of YLD and ASYR trends in China. **C** Gender-stratified analyses of YLD and ASYR trends in global. *YLD* Years Lived with Disability, *ASYR* Age-standardized YLD Rate

Comparative analysis of age-standardized YLD rate (ASYR) trends (Fig. 1A) revealed divergent epidemiological trajectories. In China, the ASYR initially declined from 1990 to 1994, reaching a nadir of 22.81 per 100,000 population (95% UI: 2.76–46.55) in 1994, followed by a sustained upward trajectory culminating in a peak of 52.70 per 100,000 (95% UI: 5.41–110.10) by 2021. In contrast, the global ASYR exhibited a monotonic increase throughout the observation period, attaining its highest recorded value of 97.66 per 100,000 (95% UI: 9.78–204.00) in 2021. Both China and the global population demonstrated significant escalations in absolute YLD counts during this interval.

### Gender disparities in high BMI-related low back pain burden between China and the world (1990–2021)

Gender-stratified analyses (Fig. 1B, C) demonstrated that females consistently exhibited a higher burden of YLDs as compared to males in both China and globally. In China, the ASYR among females reached its nadir in 1994 (28.54 per 100,000 population; 95% UI: 3.43–57.49), followed by a progressive ascent to 67.51 per 100,000 (95% UI: 6.96–142.32) by 2021. Among males, the ASYR displayed a trough in 1992 (16.93 per 100,000; 95% UI: 2.08–34.89) and peaked in 2021 at 37.45 per 100,000 (95% UI: 3.81–77.55). Globally, the ASYR for both genders demonstrated a steady upward trajectory from 1990 to 2021 (Supplementary Table S1).

### Joinpoint analysis of ASYR disparities between China and the world (1990–2021)

**Fig. 2:**
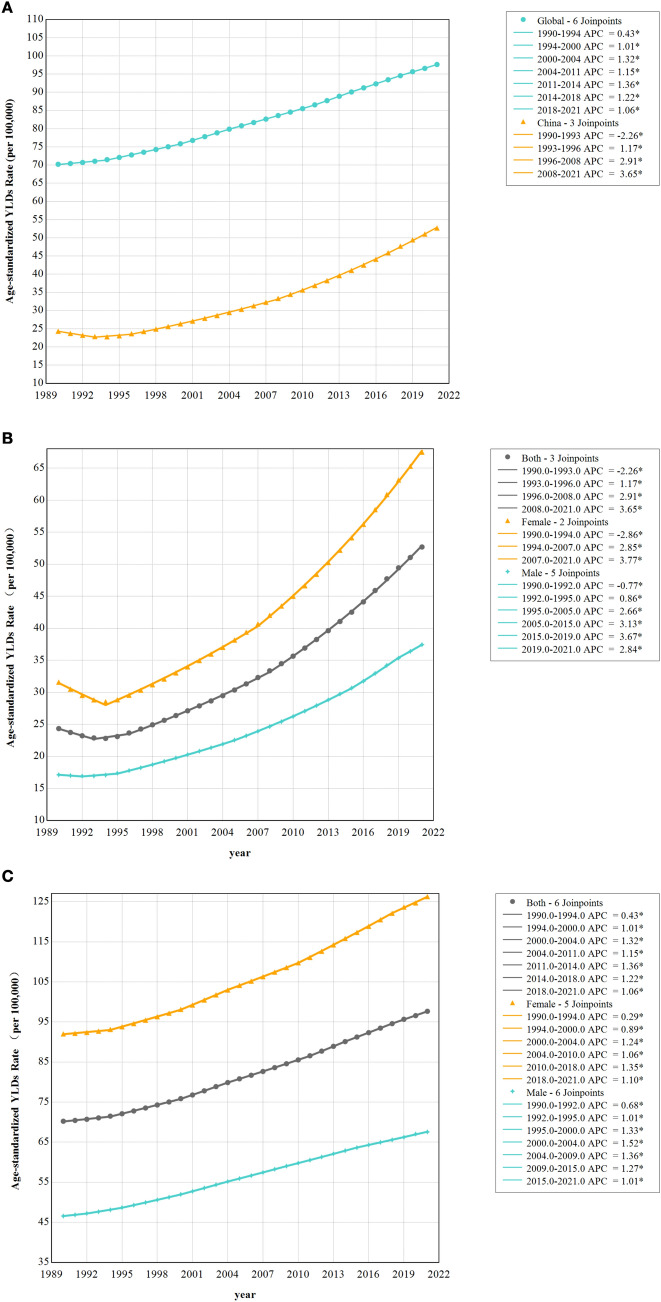
Joinpoint Analysis of ASYR Disparities between China and the world (1990–2021). **A** Joinpoint analysis of YLD and ASYR trends between China and the world. **B** Joinpoint analysis of Gender-stratified analyses of YLD and ASYR trends in China. **C** Joinpoint analysis of Gender-stratified analyses of YLD and ASYR trends in global. *YLD* Years Lived with Disability, *ASYR* Age-standardized YLD Rate

Joinpoint regression analysis (Fig. 2A) delineated markedly divergent evolutionary trajectories in ASYR between China and global populations. China's ASYR exhibited an AAPC of 2.54% (95% CI 2.46–2.62), 2.4-fold higher than the global AAPC of 1.07% (95% CI 1.06–1.09). The temporal progression of China's ASYR was partitioned into four distinct phases: 1990–1993: Significant decline (APC = −2.26%; 95% CI −2.61 to −1.90); 1993–1996: Accelerated growth (APC = 1.17%); 1996–2008: Exponential escalation (APC = 2.91%); 2008–2021: Sustained acceleration (APC = 3.65%).

In contrast, the global ASYR demonstrated stable linear growth, peaking at an APC of 1.32% during 2000–2004, lacking China's phased acceleration pattern.

Gender-stratified analyses (Fig. 2B, C) uncovered nuanced epidemiological dynamics. Among Chinese females, the ASYR followed a V-shaped trajectory, characterized by: A decline from 1990 to 1994 (APC = −2.86%); Subsequent resurgence (1994–2007: APC = 2.85%); Accelerated growth post-2007 (APC = 3.77%).

Males exhibited oscillatory increases, with peak growth during 2015–2019 (APC = 3.67%). Notably, China's AAPC values for the total population, females, and males surpassed global benchmarks by 2.37-, 2.44-, and 2.11-fold, respectively (*P* < 0.05 for all comparisons). Persistent gender disparities were evident, with females consistently enduring higher disability burdens than males (Supplementary Table S2).

### Age-specific disability burden patterns between China and the world (1990–2021)

**Fig. 3:**
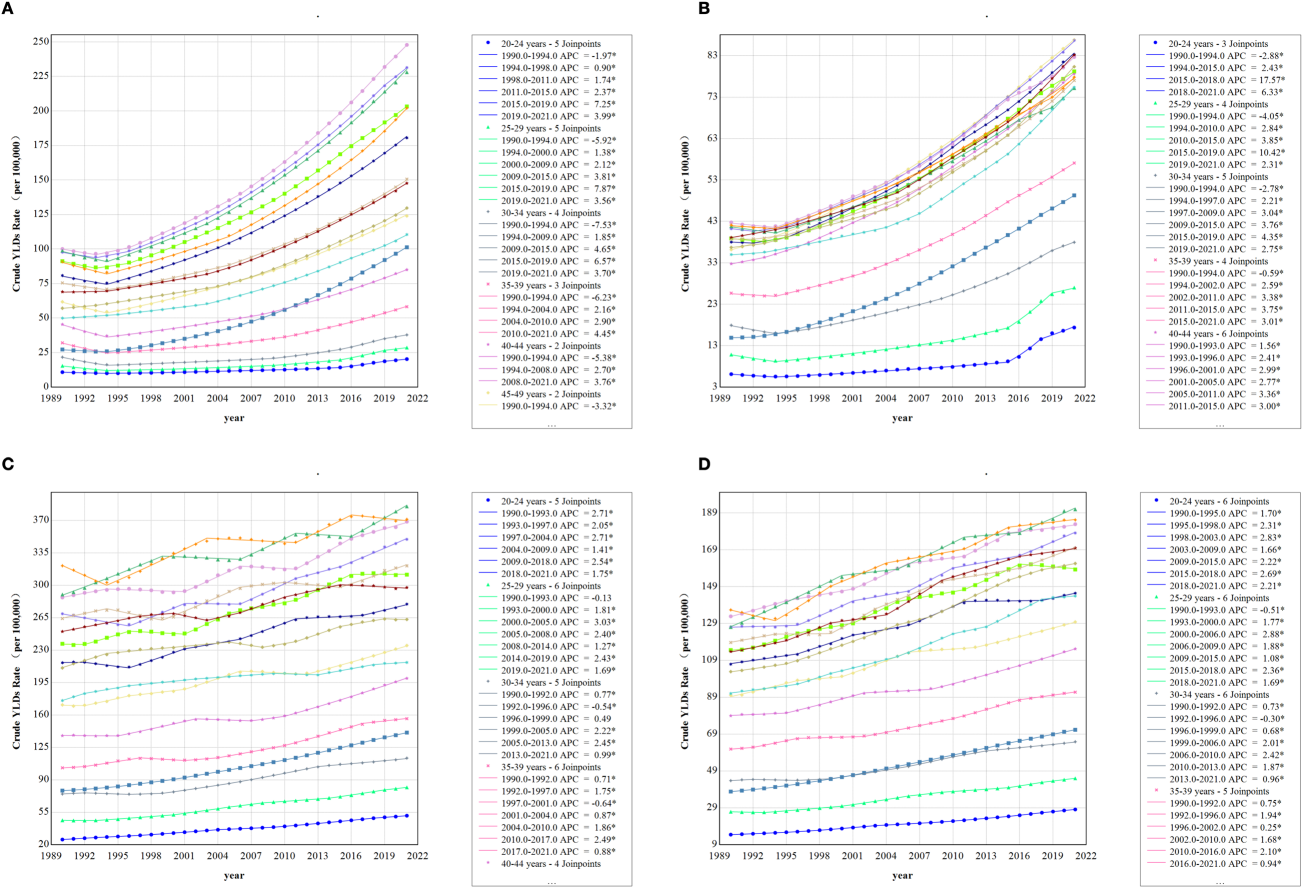
Age-Specific Disability Burden Patterns between China and the world (1990–2021). **A** Age-stratified analyses revealed distinct sex-specific patterns in YLD in China Female. **B** Age-stratified analyses revealed distinct sex-specific patterns in YLD in China Male. **C** Age-stratified analyses revealed distinct sex-specific patterns in YLD in Globle Female. **D** Age-stratified analyses revealed distinct sex-specific patterns in YLD in Globle Male. *YLD* Years Lived with Disability, *CYR* Crude YLD Rates, *APC* annual percentage change

Age-stratified analyses (Fig. 3A, B) revealed distinct sex-specific patterns in YLD rates. Among Chinese females, YLD rates demonstrated age-progressive escalations, whereas males aged 20–40 years exhibited age-dependent increases, followed by heterogeneous fluctuations beyond 40 years. Notably, since 2015, Chinese youth (20–29 years) experienced significant accelerations in YLD rates, with males showing disproportionately higher growth (e.g., 20–24 years: annual percentage change [APC] = 17.57% in males vs. 7.25% in females; 2015–2019). In contrast, global age-specific trends maintained gender-homogeneous trajectories (Fig. 3C, D; Supplementary Table S2).

### Trends in YLDs attributable to high BMI-related low back pain between China and the world (1990–2021)

**Fig. 4:**
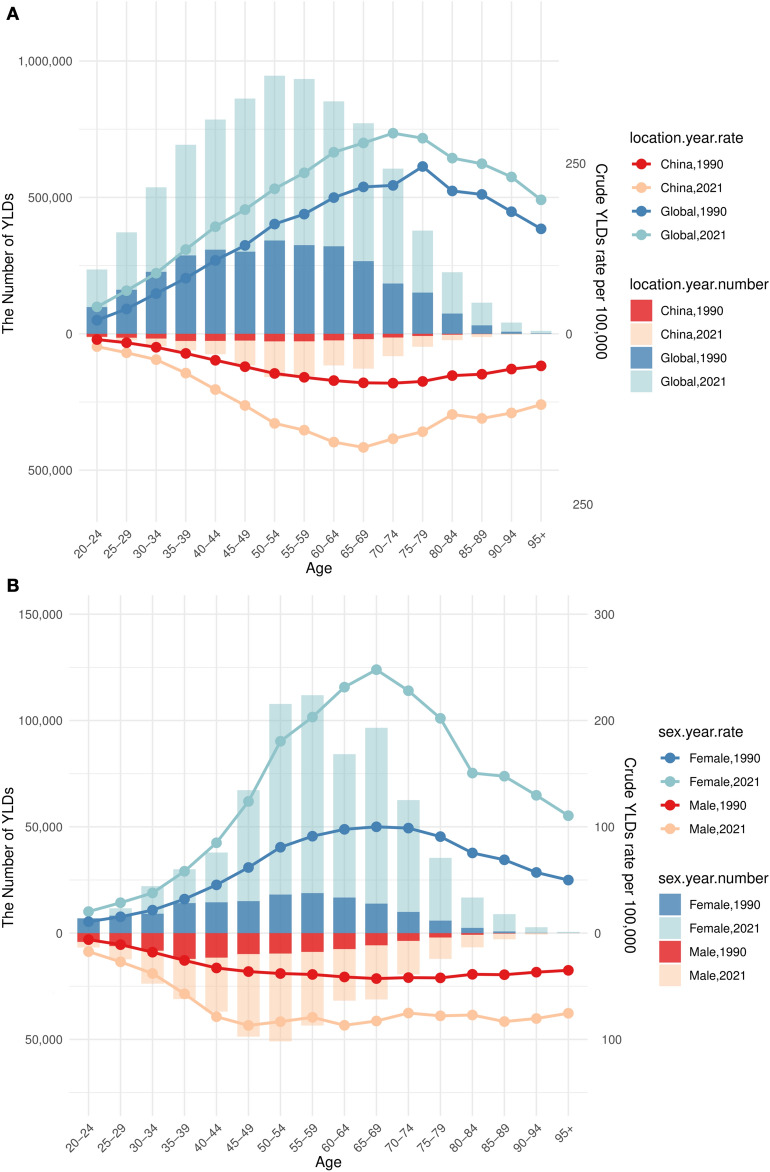
Trends in YLDs Attributable to High BMI-Related Low Back Pain between China and the world (1990–2021). **A** Analysis of YLDs and CYR across age groups between China and the global population. **B** Analysis of YLDs and CYR across Sex-Specific groups between Female and Male population in China. *YLD* Years Lived with Disability, *CYR* Crude YLD Rates

A comparative analysis of YLDs and crude YLD rates (CYR) across age groups in 1990 and 2021 revealed distinct epidemiological patterns between China and the global population (Fig. 4A). In China, YLD counts in 2021 exhibited an age-dependent increase, peaking in the 50–54-year age group (158,696; 95% UI: 16,544–356,472), whereas the global peak occurred in the same age group (946,039; 95% UI: 101,915–2,043,367), followed by a gradual decline.

China’s CYR in 2021 rose from 18.72 per 100,000 population (95% UI: 1.88–39.98) in the 20–24-year age group to a peak of 166.57 per 100,000 (95% UI: 18.09–367.51) in the 65–69 year age group, subsequently decreasing to 103.83 per 100,000 (95% UI: 10.60–214.86) among individuals aged ≥ 95 years. Globally, the CYR increased from 39.46 per 100,000 (95% UI: 3.62–86.29) in the 20–24-year age group to a peak of 294.13 per 100,000 (95% UI: 30.22–591.86) in the 70–74-year age group, declining to 196.43 per 100,000 (95% UI: 18.72–402.06) in the ≥ 95-year age group. Notably, CYR values across all age groups for both China and the global population were significantly higher in 2021 as compared to 1990 (Supplementary Table S3).

### Age- and sex-specific trends in YLDs attributable to high BMI-related low back pain in China (1990–2021)

Analysis of China’s disease burden data from 1990 to 2021 demonstrated marked increases in YLDs attributable to high BMI-related LBP, with significant heterogeneity across age and sex groups (Fig. 4B). Absolute YLD counts rose in all age groups. Older females bore a disproportionately high burden: for instance, the CYR among females aged 65–69 years surged from 100.02 to 247.84 per 100,000 population, while females aged ≥ 95 years experienced a twofold increase in CYR (49.91–110.47 per 100,000).

Conversely, younger males exhibited accelerated growth rates, with CYR among males aged 20–24 years increasing by 183.82% (from 6.12 to 17.37 per 100,000). Gender disparities widened with age, as evidenced by females aged 60–64 years having a CYR 2.67-fold higher than males (231.39 vs. 86.71 per 100,000; Supplementary Table S3).

### Projected burden of high BMI-related low back pain (2022–2036)

**Fig. 5:**
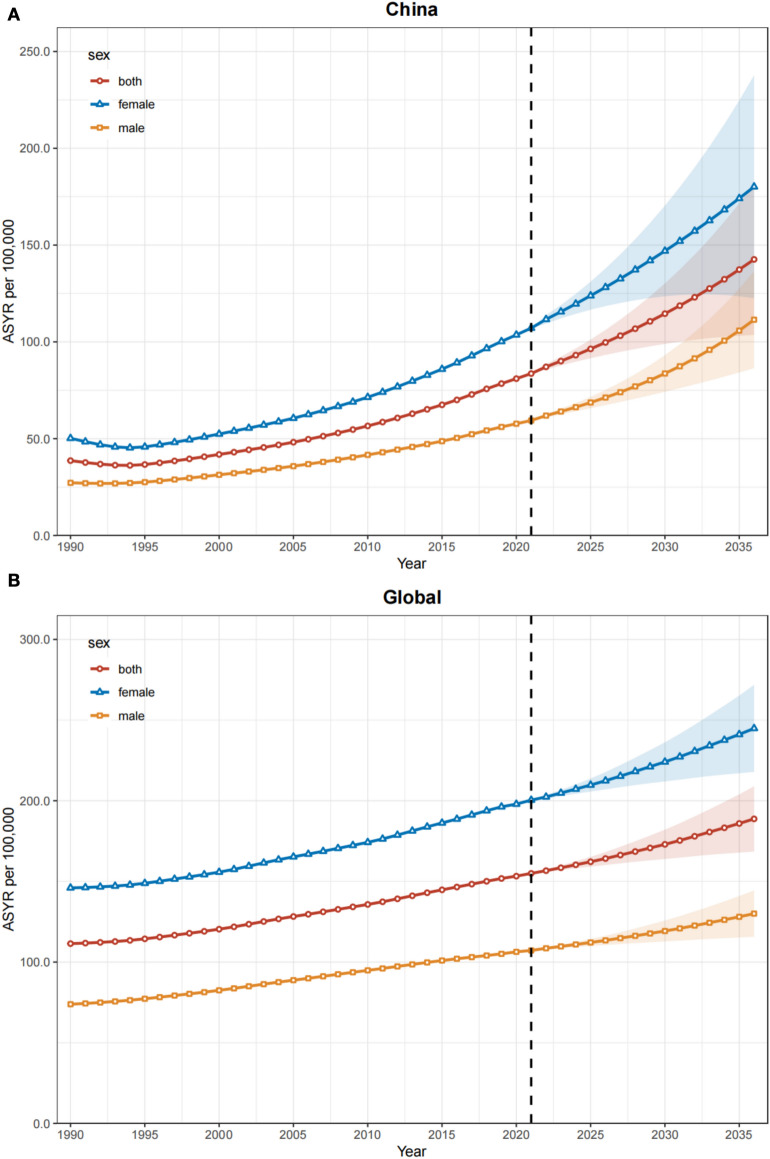
BAPC model show trends in ASYR attributable to high BMI-related LBP between China and the world (2022–2036). **A** BAPC model show trends in ASYR Attributable to High BMI-Related LBP in China. **B** BAPC model show trends in ASYR Attributable to High-BMI-Related LBP in China. *LBP* Low Back Pain, *BMI* Body Mass Index, *YLD* Years Lived with Disability, *ASYR* Age-standardized YLD Rate, *BAPC* Bayesian Age–Period Cohort

Using the BAPC model, we projected temporal trends in the age-standardized years lived with disability (YLD) rate (ASYR) of low back pain (LBP) attributable to elevated body mass index (BMI) in China and globally from 2022 to 2036 (Fig. 5; Supplementary Table S4).

In China, the ASYR for elevated BMI-associated LBP is projected to rise markedly, increasing from 83.62 per 100,000 population in 2021 to 142.60 per 100,000 by 2036—a 70.53% cumulative growth over 15 years (Fig. 5A). Sex-stratified analyses revealed consistent upward trajectories in both sexes, although females exhibited significantly higher rates, with the female ASYR projected to reach 1.62-fold higher than males by 2036.

Globally, the ASYR for high BMI-related LBP also demonstrated a sustained upward trajectory, rising from 154.97 to 188.76 per 100,000 population between 2021 and 2036, reflecting a 21.80% increase (Fig. 5B). Similarly, female rates consistently surpassed male rates, with the female ASYR expected to be 1.88-fold higher than males by 2036.

Comparative analyses revealed that China’s disease burden growth for high BMI-related low back pain substantially exceeded global trends, with the age-standardized YLD rate (ASYR) increasing by 70.53% in China versus 21.80% globally during the projection period (2021–2036). Although China’s ASYR in 2021 was 46.04% lower than the global average (83.62 vs. 154.97 per 100,000 population), this disparity is projected to narrow to 24.45% by 2036 (142.60 vs. 188.76 per 100,000), highlighting a pronounced convergence trend driven by accelerated metabolic–mechanical risk escalation.

## Discussion

This study provides the first comprehensive analysis of temporal trends in LBP attributable to elevated BMI in China from 1990 to 2021, contextualized against global benchmarks. The findings demonstrate that while China’s ASYR for high-BMI-related LBP remained significantly lower than the global average (52.70 vs. 97.66 per 100,000 population in 2021), its average annual percentage change (AAPC) of 2.54% (95% CI 2.46–2.62) markedly exceeded the global growth rate (AAPC = 1.07%; 95% CI 1.06–1.09). Notably, females exhibited persistently higher ASYRs than males across all age groups. Age-stratified analyses revealed polarized burden patterns: Youth acceleration: Young males aged 20–24 years experienced the most rapid escalation, with an APC of 17.57% (2015–2019); Geriatric predominance: Females aged 65–69 years accounted for the highest absolute burden, with CYR peaking at 247.84 per 100,000 population. The prediction based on the BAPC model further revealed that if the current trend continued, China's ASYR would surge from 83.62/100 000 in 2021 to 142.6/100 000 in 2036 (an increase of 70.53%), which was 3.24 times the global forecast for the same period (21.80%). Despite China’s overall LBP burden remaining below global levels, the accelerated growth trajectory and age–sex heterogeneities underscore the imperative for stratified public health interventions, particularly targeting postmenopausal female and young adults with elevated metabolic-spinal risks.

China has demonstrated substantial progress in mitigating LBP-related disability, paralleling improvements observed in the United States, Iran, and other nations [23, 24]. This is evidenced by a significant decline in age-standardized DALY rates (AAPC = −0.68%; 1990–2019), surpassing the global average (AAPC = −0.38%) [25]. This achievement is attributable to advancements in healthcare infrastructure, widespread implementation of occupational safety protocols, and industrial automation, which collectively reduced mechanical spinal injury incidence [26]. However, our study identifies an emerging public health paradox: accelerating BMI-driven LBP risks, driven by escalating obesity rates.

China currently faces a severe obesity epidemic, with projections indicating that 70% of adults may be overweight or obese by 2030 if current trends persist [26]. A multifactorial cohort analysis from the UK Biobank highlights obesity as the predominant risk factor for multimorbidity, exceeding the impact of low socioeconomic status, suboptimal diet, smoking, alcohol misuse, or physical inactivity [27]. Mechanistically, obesity exacerbates LBP pathogenesis through epigenetic dysregulation, oxidative stress, and sterile inflammation, which collectively impair immune homeostasis, accelerate musculoskeletal aging, and amplify risks of chronic noncommunicable diseases [28–30].

Modern sedentary behaviors further potentiate these risks: prolonged sitting not only reduces energy expenditure, promoting adiposity, but also induces disuse atrophy of paraspinal musculature, destabilizing spinal biomechanics [31]. The synergistic convergence of obesity-related metabolic dysfunction and sedentariness-induced mechanical compromise creates a bidirectional pathway for LBP exacerbation. To sustain progress in overall disability reduction, China must prioritize integrated strategies targeting high BMI risks, including metabolic health promotion, ergonomic workplace interventions, and public awareness campaigns addressing sedentary lifestyles.

Joinpoint regression analysis of ASYR from 1990 to 2021 identified critical challenges in managing high-BMI-related LBP in China. The ASYR increased at an annualized rate of 2.54% (95% CI 2.46–2.62), 2.37-fold faster than the global average (1.07%; 95% CI 1.06–1.09). This disparity is attributed to synergistic urban lifestyle transitions, including reduced physical activity, dietary shifts toward energy-dense foods, and prolonged occupational sedentariness. Notably, China’s ASYR trajectory followed a four-phase progression: 1990–1993: Decline (APC = −2.26%; 95% CI −2.61 to −1.90); 1993–2008: Accelerated growth (APC = 1.17–2.91%); 2008–2021: Sustained escalation (APC = 3.65%; 95% CI 3.53–3.77); The post-2008 acceleration aligns temporally with China’s national obesity epidemic, which surged after 2006 [32], suggesting a dose-dependent cumulative effect of BMI-related metabolic disturbances on LBP pathogenesis.

The BAPC prediction model quantified the temporally dependent relationship between obesity exposure and LBP burden. The analysis suggests that prolonged obesity exposure may drive nonlinearly accelerated progression of metabolic disorders and intervertebral disc degeneration, disproportionately impacting rapidly developing economies. China’s LBP burden growth rate—2.3-fold higher than global trends—reflects unique challenges arising from its rapid economic transition: occupational sedentariness [33] (e.g., manufacturing automation), digital economy-driven screen dependency [34] (e.g., adolescent electronic device overuse), and dietary transitions synergistically amplify obesity-related metabolic–mechanical risks. Critically, observed LBP burden surpassed conventional epidemiological thresholds [35], underscoring the inadequacy of existing prevention frameworks.

Gender-stratified analyses revealed persistently higher ASYR among females, characterized by a distinct "V-shaped" trajectory with peak growth rates of 3.77% (95% CI 3.65–3.89), marginally exceeding male rates (3.67%; 95% CI 3.55–3.79), a pattern consistent with global trends. This divergence likely arises from sex-specific biological and biomechanical mechanisms: younger females face elevated LBP risks during pregnancy due to increased lumbar mechanical loading and adaptive spinal curvature changes (e.g., augmented lumbar lordosis) [36], while postmenopausal female experience accelerated bone mineral density loss and paraspinal muscle atrophy linked to estrogen decline, exacerbating spinal instability [37]. In contrast, although males exhibited lower overall burden, young male aged 20–24 demonstrated alarming YLD rate growth (APC = 17.57%; 95% CI 15.32–19.82; 2015–2019), likely attributable to occupational stress-induced circadian disruption and prolonged screen-based sedentariness [31, 38] Notably, the stabilization of male burden after age 40 may reflect the efficacy of labor protection policies (e.g., ergonomic workplace regulations) and chronic disease management programs targeting metabolic and musculoskeletal health [39].

This study revealed significant regional disparities in the burden of low back pain attributable to high BMI. The most severely affected regions encompassed Central/Eastern Europe/Central Asia and high-income areas. Despite their advanced economic status and healthcare infrastructure, these regions exhibited aggravated obesity-related LBP burdens due to high-calorie dietary patterns and sedentary occupational environments [40]. Notably, the highest growth rates of BMI-related LBP burden were observed in developing regions, particularly South Asia, Southeast Asia, and China, with AAPC reaching 2.54 and 2.79 in China and South Asia respectively. These rapidly developing regions are undergoing substantial lifestyle transitions during economic urbanization. In China, for instance, the proliferation of Western fast-food culture has altered traditional dietary structures, while technological advancements have transformed labor patterns towards reduced physical activity and increased sedentary behaviors. Although China demonstrates relatively low age-standardized YLD rates (ASYR), its substantial absolute YLD numbers underscore the compounding effects of population aging on BMI-related LBP burden.To address these challenges, a multipronged intervention strategy is proposed: 1. Targeted screening and early intervention for high-risk populations; 2. Nationwide promotion of healthy lifestyles coupled with institutional weight management clinics; 3. Development of stratified care models addressing the "overweight-LBP-disability" triad through multidisciplinary collaboration across medical, psychological, and social domains [41]. These evidence-based recommendations aim to mitigate the accelerating epidemic of BMI-related LBP and advance the strategic objectives of the "Healthy China 2030" initiative through coordinated health policy reforms and innovative care delivery systems [42].

## Limitations

This study has several limitations. First, Reliance on GBD secondary data may introduce spatiotemporal biases, including regional disparities in data collection (e.g., model-based estimates in low-income countries), self-reporting biases in BMI measurements, heterogeneity in LBP diagnostic criteria, unadjusted confounders (e.g., occupational factors), and temporal data gaps. Second, the assumption of uniform LBP severity across healthcare intervention contexts lacks clinical granularity [43]. Third, the macrolevel analysis limits generalizability to specific subgroups or regions within China and globally. Future research should prioritize subnational analyses (e.g., provincial-level assessments in China) and incorporate additional risk factors (e.g., occupational ergonomics, genetic predispositions) to refine LBP prevention frameworks. Finally, the exclusion of individuals aged < 20 years underscores the need for longitudinal studies examining early-life BMI trajectories and their association with adolescent LBP onset, which could inform primordial prevention strategies.

## Conclusion

China exhibits declining overall LBP burden but rising obesity-related LBP, with ASYR growing faster than global averages. The BAPC model projects that China's disease burden will increase at a growth rate significantly exceeding the global average in the coming decades. Persistent gender (female predominance) and age disparities (youth male escalation) highlight stratified risk profiles. Prioritizing sex- and age-targeted interventions, alongside metabolic–mechanical pathway modulation, is critical to curb this dual burden.

## Supplementary Information

Supplementary material 1.

## Data Availability

No datasets were generated or analysed during the current study.

## Abbreviations

LBP: Low back pain
BMI: Body mass index
GBD: Global burden of disease
DW: Disability weight
CI: Confidence interval
YLD: Years lived with disability
DALY: Disability-adjusted life year
SPC: Simple percentage change
APC: Annual percentage change
AAPC: Average annual percentage change
ASYR: Age-standardized YLD Rate
CYR: Crude YLD Rates
BAPC: Bayesian age–period cohort
UI: Uncertainty interval
SEL: Spinal epidural lipomatosis
